# Computational NMR Study of Benzothienoquinoline Heterohelicenes †

**DOI:** 10.3390/ijms25147733

**Published:** 2024-07-15

**Authors:** Valentin A. Semenov, Gary E. Martin, Leonid B. Krivdin

**Affiliations:** 1 A.E. Favorsky Irkutsk Institute of Chemistry, Siberian Branch of the Russian Academy of Sciences, Favorsky St. 1, 664033 Irkutsk, Russia; krivdin55@gmail.com; 2 Department of Chemistry and Biochemistry, Seton Hall University, 400 South Orange Ave., South Orange, NJ 07079, USA

**Keywords:** benzothienoquinoline heterohelicenes, ^1^H NMR, ^13^C NMR, molecular geometry

## Abstract

Early NMR studies of several heterohelicenes containing an annular nitrogen atom and a thiophene ring in their structure suggested the possibility of the lengthening of the carbon–carbon bonds in the interior of the helical turn of the molecule based on the progressive upfield shift of ^13^C resonances toward the center of the helical turn. We now report a comprehensive analysis of the optimized geometry and a comparison of the calculated vs. observed ^1^H and ^13^C NMR chemical shifts of nineteen representative benzothienoquinoline heterohelicenes. As was initially hypothesized on the basis of the progressive upfield shift of carbon resonances toward the center of the interior helical turn, the present computational study has demonstrated that carbon–carbon bonds indeed have more *sp3* character and are longer than normal *sp*^2^ bonds to accommodate the helical twist of the molecule, as expected.

## 1. Introduction

During the 1980s and early 1990s, one of the authors was involved in a series of NMR studies of thiophene-containing compounds that included the mutagen phenanthro[3,4-*b*] thiophene [1], benzo[*b*]phenanthro[4,3-*d*]thiophene [2], beno[*f*][1]benzthieno[2,3-*c*]- quinoline [3], and [1]benzothieno[2,3-*c*]naphtho[1,2-*f*]quinoline [4], which are analogs of classical hexahelicene, which was first synthesized by Newman and Lednicer [5] in 1956. The precise conformation of these severely overcrowded aromatic molecules was historically reported by Mackay, Robertson, and Sime [6], who used X-ray crystallography to demonstrate the essential out-of-plane deviations of the benzene ring scaffold (known as the “helical twist”).

**Scheme 1:**
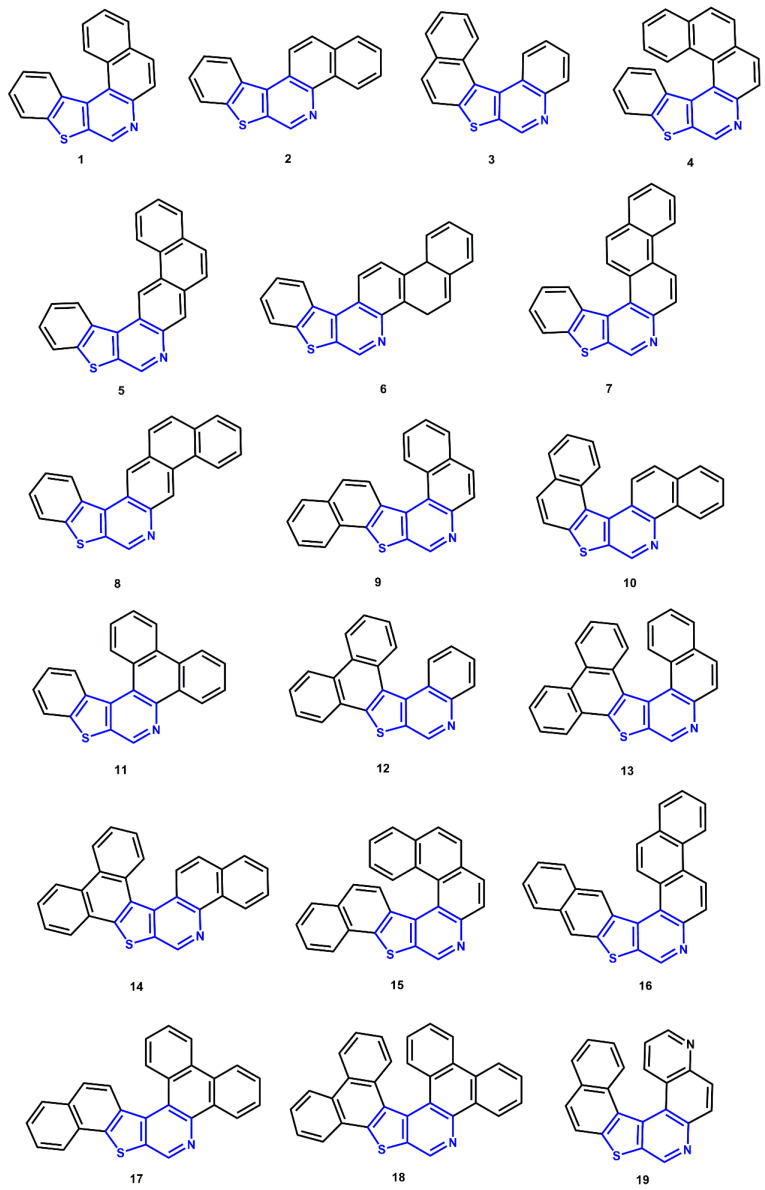
Benzothienoquinoline heterohelicenes **1**–**19** investigated in this study.

In a continuation of our preliminary communication [7], in this contribution, we have investigated the question of the length of the carbon–carbon bonds in the interior of the helical turn of benzothienoquinoline heterohelicenes in conjunction with a comparison of the assigned and calculated ^1^H and ^13^C NMR chemical shifts for the series of nineteen representative members of this series, **1**–**19**, shown in Scheme 1 [3,4,5,8,9,10,11,12,13,14,15,16,17,18,19]. This study was prompted by an early observation that the ^13^C shift of non-protonated carbons in the interior of the helical turn of heterohelicenes shifted progressively upfield as molecules became larger and hence more helical in character, which suggested the potential lengthening of the carbon–carbon bonds to accommodate the helical twist of the molecule. Since polynuclear heteroaromatics have highly congested ^13^C spectra despite the significantly greater dispersion of ^13^C spectra relative to the corresponding ^1^H spectral range, determining additional criteria that can serve to facilitate spectral assignment can be highly beneficial. This is particularly true since prior to the communication of the i-HMBC [20] experiment in the spring of 2023, there was no definitive way to differentiate ^2^*J*_CH_ from ^3^*J*_CH_ correlations in routine HMBC spectra. However, if there is a well-documented and confirmed upfield shift of carbons in the interior of the helical turn of heterochelicenes relative to carbons on the perimeter of the helix, assignments can be made with more confidence, potentially avoiding spectral or even structural misassignments.

**Figure 1:**
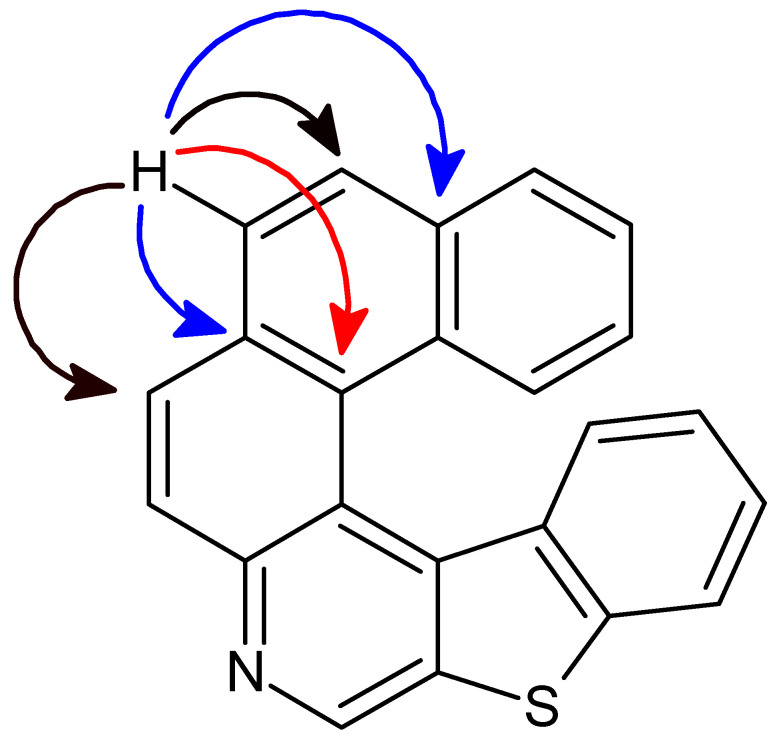
Example of HMBC correlations for one of the protons in heterohelicene **4**. Two- and three-bond heteronuclear correlations to protonated carbons on the exterior of the helicene are designated by black arrows; two- and three-bond correlations to non-protonated carbons on the exterior of the helix are designated with blue arrows; the single three-bond correlation from the example proton to a non-protonated carbon on the interior of the helical turn is designated by the red arrow. As shown computationally in this report, correlations via two- and/or three-bonds to non-protonated carbons on the exterior perimeter of the molecule will be observed to carbons resonating downfield of the carbon correlated via three-bonds on the interior of the helical turn, providing an additional assignment criterion.

As an illustration of the point made in the previous paragraph, consider the example afforded by **4** shown in Figure 1. Obviously, three-bond HMBC correlations to protonated carbons can be easily differentiated from correlations to non-protonated carbons on the basis of relaxation efficiency considerations even without performing T_1_ relaxation measurements. However, differentiating a two-bond correlation to a non-protonated carbon on the exterior of the helical turn from a three-bond correlation to a carbon on the interior of the helix is less straightforward. This is where knowledge of the behavior of carbon chemical shifts on the interior of the helical turn becomes beneficial as a potential assignment criterion.

## 2. Computational Details

Geometry optimizations of **1**–**19** were performed with the Gaussian 09 [21] suite of programs at the M06-2X/cc-pVTZ//aug-cc-pVTZ level (diffuse functions were used only on nitrogen and sulfur atoms to account for the effect of the multiple diffused lone pairs). The solvent effect was accounted for within the Integral Equation Formalism Polarizable Continuum Model (IEF-PCM) [22,23]. The optimized structures of **1**–**19** were found to be the true minima on the potential energy surface, as was demonstrated by the absence of imaginary frequencies at that computational level. The corresponding Cartesian coordinates of the optimized benzothienoquinoline heterohelicenes **1**–**19** are given in the Supporting Information. We have carried out an additional study of geometric parameters calculated with different DFT functionals for a representative molecule of [1]benzothieno[2,3-*c*]naphtho[1,2-*f*]quinoline (**4**). It turned out that the choice of the DFT method does not have a noticeable effect on the discussed trends of the C-C bond lengths while passing from the internal turn of the helix to the external one. The principal conclusion remains the same, namely that steric strain of the inner turn leads to elongation of the corresponding C-C bonds. The results of this block of calculations are given in the SI, see Table S2.

All calculations of ^1^H and ^13^C NMR isotropic magnetic shielding constants (the latter being converted into chemical shifts) of **1**–**19** were carried out at the DFT level in the liquid phase by applying the Gaussian 09 [21]. In these calculations, we used the functional of Perdew, Burke, and Ernzerhof [24,25] with a predetermined amount of the exact exchange, with the latter known as PBE0 [26]. This functional was used here within the recently proposed pecS-2 basis set, which was specially developed for calculating shielding constants in large synthetic compounds and natural products [27,28]. This basis set makes it possible to achieve better correlation with the experimental data (and, accordingly, higher accuracy) at an acceptable computational resource cost.

Calculated proton and carbon isotropic magnetic shielding constants of **1**–**19** were then converted into ^1^H and ^13^C NMR chemical shifts, as recommended by the International Union of Pure and Applied Chemistry (IUPAC) [29]. To account for systematic errors of calculated chemical shifts, we have established correlations between their isotropic magnetic shielding constants (*y*) and experimental chemical shifts (*x*). Resulting linear regressions were further used to define the equations of the y = *a*x + *b* type. The slope *a* and intercept *b* were then used for recalculating the unscaled shielding constants (*σ*_calc_) into the scaled values of chemical shifts (*δ_s_*) as *δ_s_* = (*σ_calc_* – *b*)/*a*. Linear regression parameters of correlation plots of **1**–**19** are given in SI, see Table S1. Complete data set of calculated ^1^H and ^13^C NMR chemical shifts are available at the SI.

## 3. Results and Discussion

### 3.1. Bond Lengths

**Figure 2:**
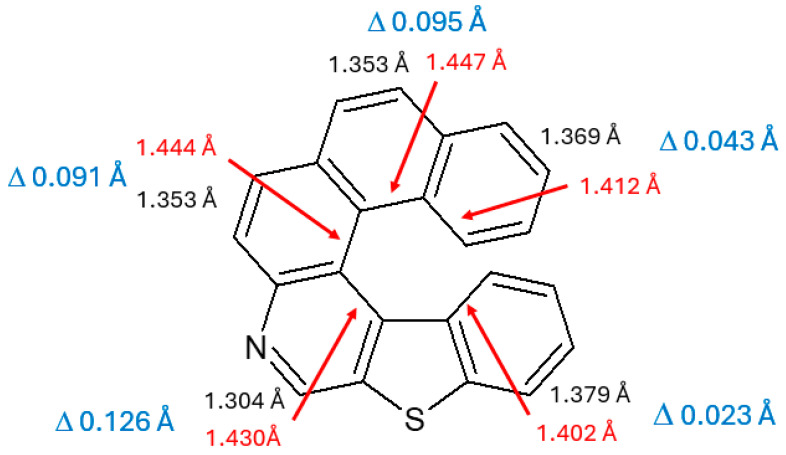
Structure of **4** contrasting bond lengths of protonated carbon–carbon or protonated carbon–nitrogen pairs on the exterior of the helical molecule (bond distances shown in black) with the non-protonated carbon–carbon bond lengths on the opposite “face” of the phenyl ring on the interior of the helical turn (shown in red; ΔÅ values are shown in blue).

Molecular geometry optimization of **1**–**19** was performed at the M06-2X/cc-pVTZ//aug-cc-pVTZ level in chloroform. First, it should be noted that when carbon–carbon bond lengths between adjacent non-protonated carbon pairs of a given phenyl on the interior of the helical turn of these molecules are compared with the bond length between the pair of protonated carbons on the opposite “side” of the same ring on the exterior of the molecule, there is a lengthening of the bonds on the interior of the helical turn that is necessary to accommodate the helical twist of the molecule, as expected. Consider the pairwise comparisons for **4**, which are highlighted in Figure 2.

**Figure 3:**
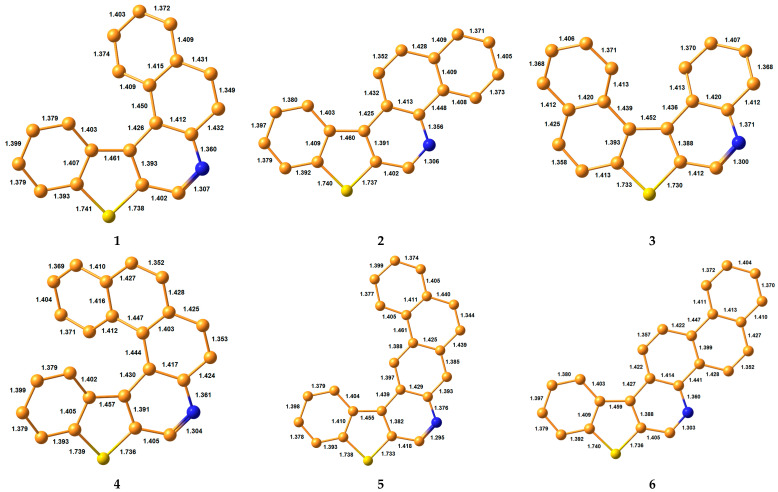

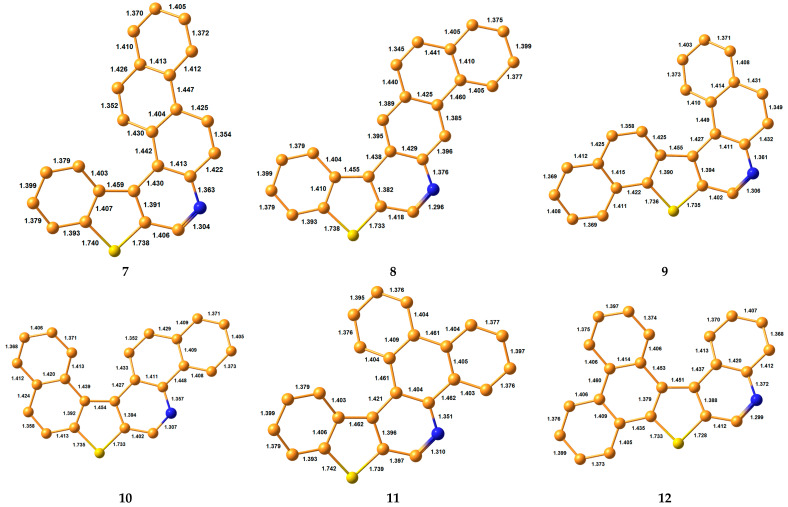

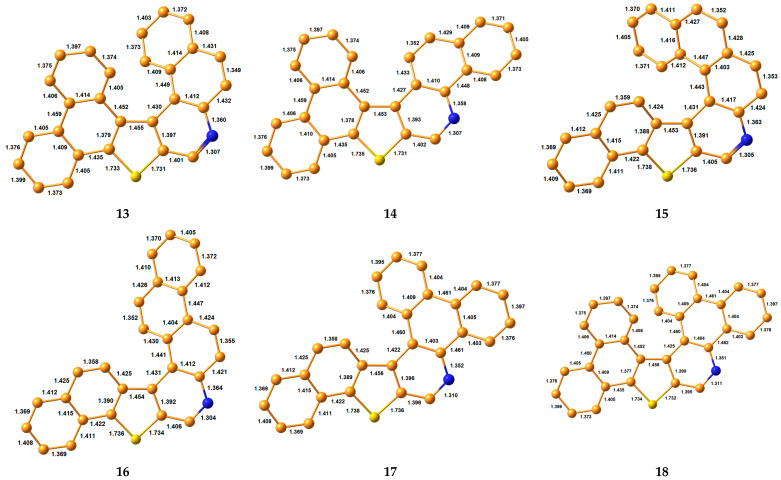

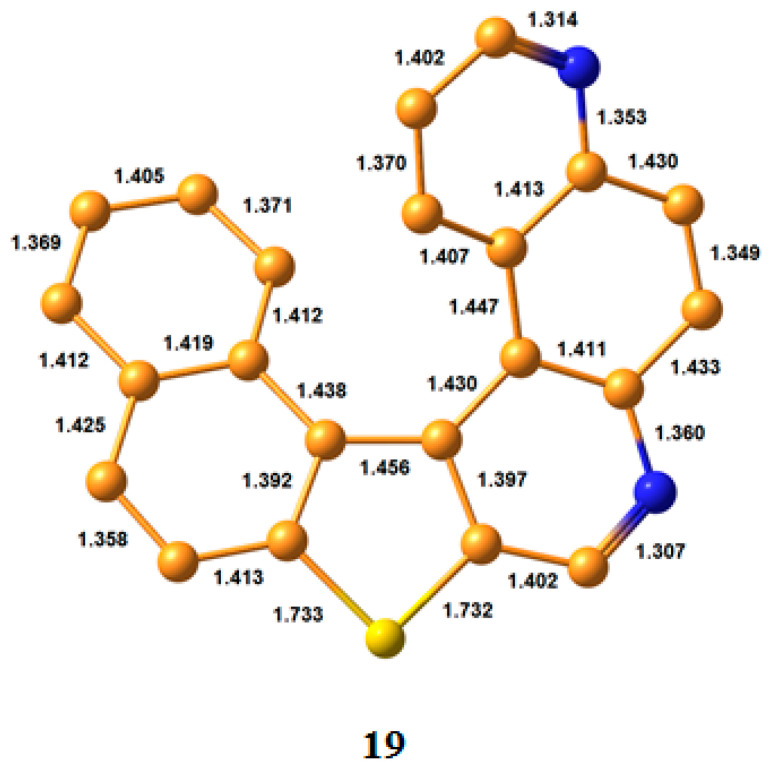
Carbon–carbon bond lengths (Å) of benzothienoquinoline heterohelicenes **1–19** optimized at the M06-2X/cc-pVTZ//aug-cc-pVTZ level in the liquid phase. Element colors: carbon - orange; nitrogen - blue; sulfur - yellow.

The phenyl ring bond differences shown in Figure 3 range from 0.023 and 0.043 Å for the two terminal phenyl rings of the helix to 0.91 and 0.95 Å for phenyl bond more toward the center of the interior turn of the helix. When the exterior atom pair involves an annular nitrogen and a carbon atom, the difference is even more pronounced at 0.126 Å, although the much shorter C=N bond length on the exterior is expected. While these are seemingly subtle differences, as is shown below, these differences nevertheless correspond to an appreciable difference in chemical shift behavior that afford investigators with an orthogonal check of assignment accuracy in those cases where more advanced NMR experimental techniques are either not available or are time- or sample-prohibitive.

### 3.2. Chemical Shifts

**Figure 4:**
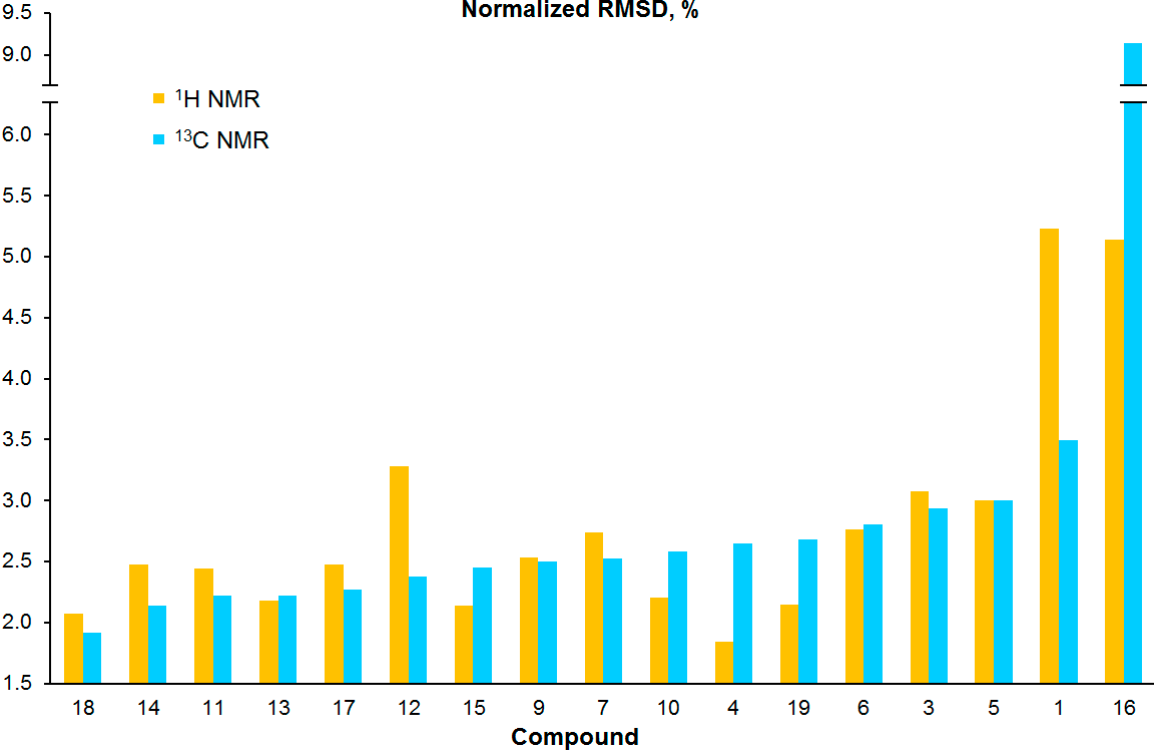
Normalized root-mean-square deviations (%) of calculated ^1^H and ^13^C NMR chemical shifts of **1**–**19**. Compounds are arranged in order of the increasing NRMSD of their ^13^C NMR chemical shifts.

**Figure 5:**
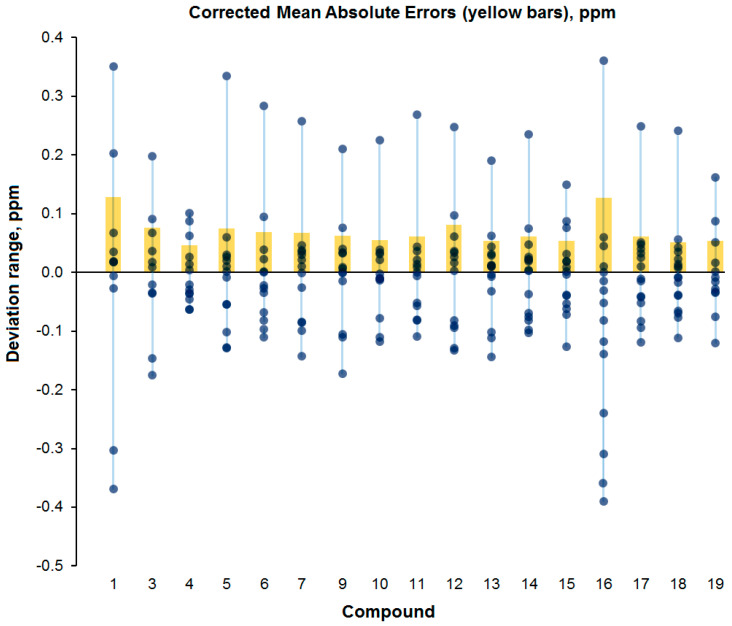
Deviation ranges of calculated ^1^H NMR chemical shifts (shown as dark blue dots) corresponding to each individual proton in each particular compound **1**–**19** together with corresponding CMAE values (given as yellow bars).

Figure 4 and Figure 5 provide calculated ^1^H and ^13^C NMR chemical shifts of benzothienoquinoline heterohelicenes **1**–**19** compared with the experimental chemical shift data. Considering all reported experimental chemical shift data together with their calculated values, a correlated estimation of chemical shifts was conducted, taking into account statistical descriptors including normalized root-mean-square deviation (NRMSD) and corrected mean absolute error (CMAE).

As is seen in Figure 4, generally a good correlation can be observed between theoretical and experimental NMR data. The RMSD values, normalized to the range of chemical shifts for each compound from this series, were found to be of about 1.8–3.0% for protons and 1.9–2.8% for carbons for most structures. In view of the fact that all heterohelicenes considered in this study include only *sp*^2^-hybridized carbons, the range of their ^13^C NMR chemical shifts is very narrow amounting to only ca. 25 ppm. If annular nitrogen was not incorporated into the structures, the range would be still narrower. The resulting NRMSD of less than 3% for the series of the studied compounds **1**–**19** indicates the effectiveness of the computational scheme employed including, in particular, the original pecS-2 basis set. However, for a wider series of organic compounds including all types of carbon atoms (namely, *sp*, *sp*^2^, and *sp*^3^), we estimate the corresponding NRMSD would not exceed 0.5%.

Returning to Figure 4, one can see that two compounds from this series, specifically **1** and **16**, stand out from the overall level of correlation. Thus, the NRMSD in the case of the ^1^H NMR data are 5.23 and 5.14%, while for the ^13^C NMR data, these values are 3.50 and 9.14% for compounds **1** and **16**, respectively. To understand these anomalous deviations in more detail, we present Figure 5, which shows the range of deviations of theoretically calculated ^1^H NMR chemical shifts from their experimental values for each of the studied compounds.

It is obvious that for most structures, the ^1^H chemical shift deviations fall within the range of −0.15 to +0.10 ppm. Moreover, almost all compounds have one point that has the largest positive deviation at a level of +0.20 to +0.35 ppm. This point corresponds to the chemical shift of the hydrogen atom on the carbon vicinal to the annular nitrogen. Based on this observation, one can conclude that this proton experiences significant anisotropic influence of the benzothienoquinoline system, which cannot be fully reproduced theoretically within the framework of the applied calculation scheme.

**Scheme 2:**
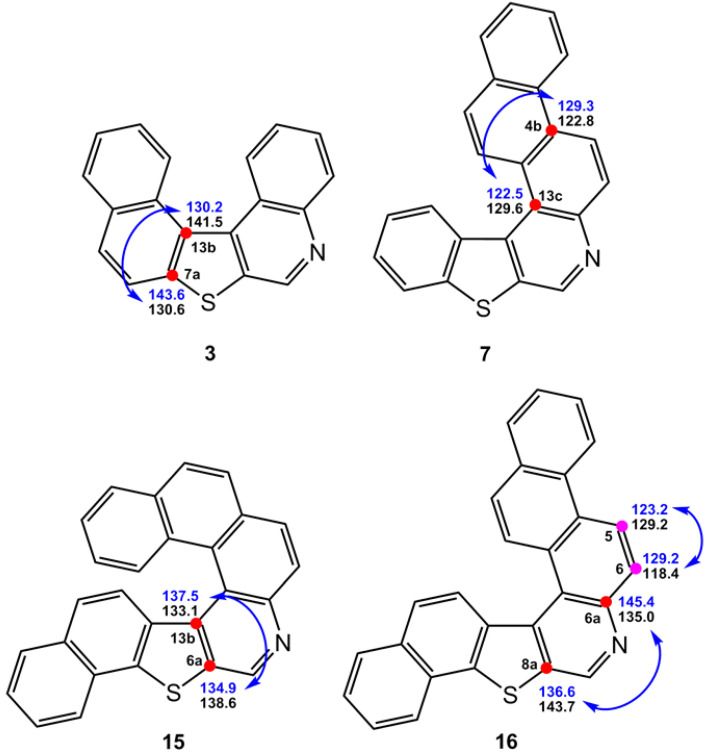
Suggested pairwise reassignments of experimental ^13^C NMR chemical shifts of compounds **3**, **7**, **15**, **16** based on the present work: black—experimental data, blue—calculated in this paper.

In addition, as it was noted earlier, significant deviations to both the low and high field are observed for compounds **1** and **16**, underscoring the probabe need for specific resonance reassignments. Based on the calculations of the NMR chemical shifts performed, some pairwise resonance reassignments of the individual signals in the ^13^C NMR spectra are proposed for these heterohelicenes (shown in Scheme 2 with blue arrows). More detailed comments on these reassignments for particular compounds of this series follow.

Naphtho[1′,2′:4,5]thieno[2,3-*c*]quinoline (**3**)—for this compound, reassignment of the ^13^C NMR signals of carbons 7a and 13b appears warranted. For [1]benzothieno[2,3-*c*]naphtho[2,1-*f*]quinoline (**7**) [10], the reassignment of the ^13^C NMR signals of 4b and 13c is proposed. In the case of naphtho [1,2-*f* ]naphtho[2′,1′:4,5]thieno[2,3*-c*]quinolone (**15**) [16], we suggest that the ^13^C NMR signals of 6a and 13b may need to be reversed. Finally, for naphtho[2,1*-f*]naphtho- [2′,1′:4,5] thieno[2,3*-c]*quinoline (**16**) [17], it seems likely that the ^13^C NMR signals of the resonances pairs for carbons 5, 6 and 6a, 8a should be swapped pairwise, as shown in Scheme 2.

**Scheme 3:**
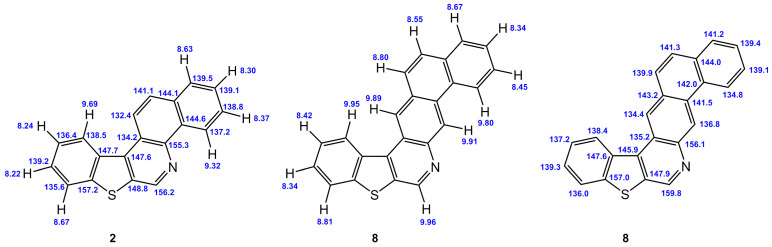
Calculated ^1^H and ^13^C NMR chemical shifts of compounds **2** and **8** (given in blue) that were not reported in the original publications based.

Further, based on the calculations performed, some experimentally unresolved and/or unreported resonances in the ^1^H and ^13^C NMR spectra of compounds **2** [8] and **8** [12] are suggested in Scheme 3.

**Figure 6:**
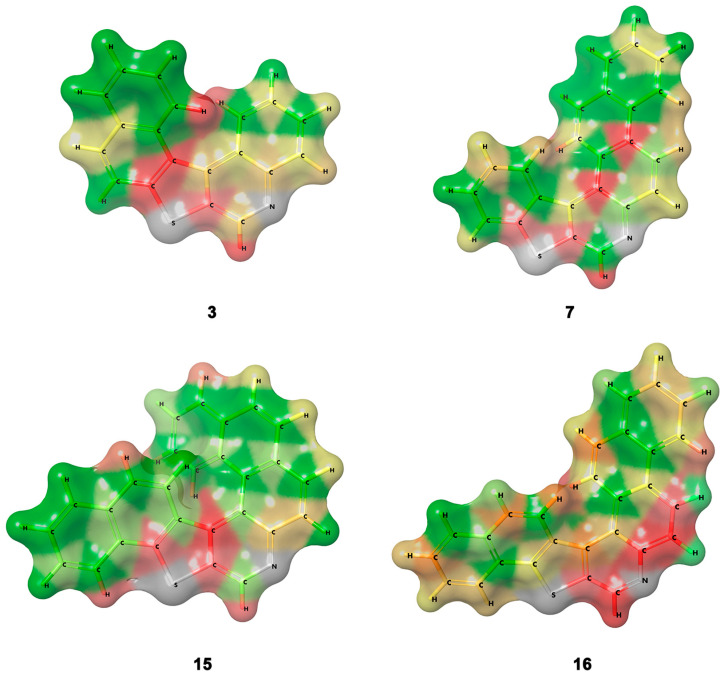
Distribution of errors for the calculation of ^1^H and ^13^C NMR chemical shifts in compounds **3**, **7**, **15**, and **16**. The color gradation from green to red corresponds to the increase in the calculation error.

Distribution of errors for the calculation of ^1^H and ^13^C NMR chemical shifts in compounds **3**, **7**, **15**, and **16** is shown graphically in Figure 6. Systematically large errors for both ^1^H and ^13^C NMR chemical shifts were observed in proximity to the thienoquinoline moiety in these congeners. This observation suggests that this structural component provides somewhat unpredictable stereoelectronic effects, which are poorly taken into account at the DFT level. However, generally, there is a good correlation between the calculations that were performed with the experimental data. Figure 6 shows obvious deviations for carbon chemical shifts (marked in red), that most probably need to be reassigned.

**Figure 7:**
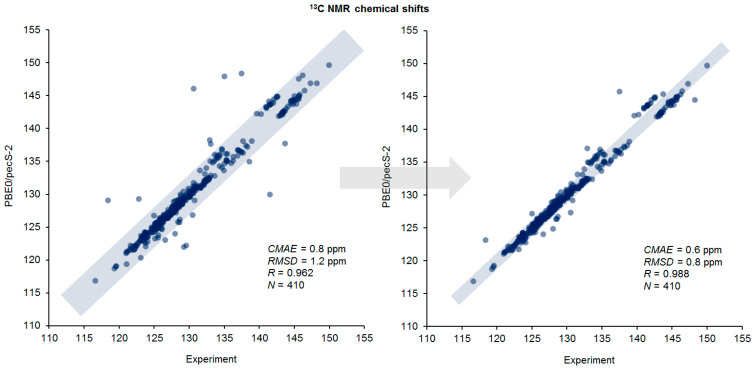
Correlation plots of the calculated vs. experimental ^13^C NMR chemical shifts of **1**–**19**: original (**left**) and reassigned (**right**).

As a final illustration of this segment of the study, a correlation plot comparison between the original and reassigned calculated ^13^C NMR chemical shifts of **1**–**19** against experiment are presented in Figure 7. Generally, there is excellent agreement between the calculated ^13^C NMR chemical shifts and the experiment data, as manifested by CMAE and RMSD errors for the original and reassigned values. The latter are, accordingly, 0.6 and 0.8 ppm, as compared with 0.8 and 1.2 ppm for the former in the range of 116–152 ppm, when 410 chemical shifts are evaluated.

As was briefly discussed in Section 3.1, the relief of steric strain in the interior of the helical turn in **1**–**19** corresponds to a slight lengthening of carbon–carbon bonds, when compared with bonds on the exterior of the helix. Based on this observation, one should expect a systematic decrease in the *s*-character of carbon *σ*-bonds in the inner helical turn.

**Figure 8:**
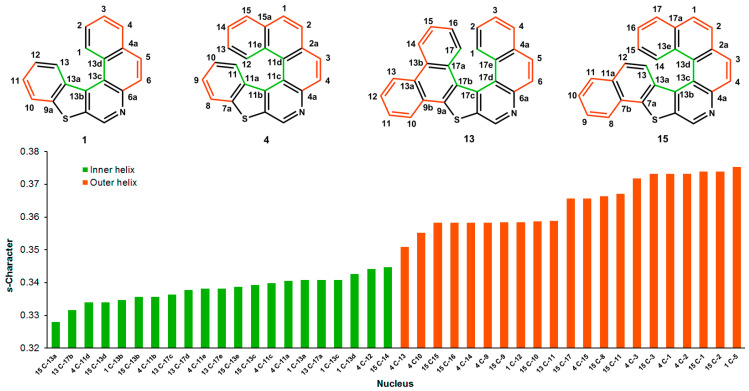
Dependence of the *s*-character of carbon–carbon bonds in the inner and outer helix of compounds **1**, **4**, **13**, and **15** on the position of nuclei.

To test this hypothesis and evaluate changes in the hybridization of carbon atoms depending on their location in either the inner or outer helical turns, a molecular analysis was carried out within the framework of the Natural Bond Orbitals (NBO) [30,31,32,33]. Based on the results of the NBO analysis performed, it was shown that, in general, the *σ*_C=C_ bonds of the outer turns of heterohelicenes have greater *s*-characters than the *σ*_C=C_ bonds of the internal turn, see Table 1 (the numbering scheme for atoms is provided in Figure 8).

The effect of the decrease in *s*-character with the increase in carbon–carbon *σ*-bond length has long been known and is well-studied. However, in the present paper, we have demonstrated a systematic manifestation of this effect in the series of helically twisted heterohelicenes. This effect is expressed mainly in the weakening of the overlap of carbon *s*-orbitals and strengthening of the overlap of *p*-orbitals, which follows from the data presented in Table 1. As a result, the hybridization of the atoms participating in this interaction changes partially from *sp^2^* to *sp*^3^ type, a graphical representation of the *s*-character for the inner and outer turns.

## 4. Conclusions

Geometry optimization of the historically attractive benzothienoquinoline heterohelicenes **1**–**19** performed at the M06-2X/cc-pVTZ//aug-cc-pVTZ level in chloroform-*d* revealed that the carbon–carbon bonds located in the interior of the helical turn of the molecule were somewhat lengthened as compared with those located in the external part of the helix, to accommodate the helical twist of the molecule, as was originally suspected. ^1^H and ^13^C NMR chemical shifts calculated at the PBE0/pcS-2 level were in excellent agreement with experiment reflecting the progressive upfield shift of ^13^C resonances toward the center of the helical turn.

A detailed examination of the error distribution for the calculation of ^1^H and ^13^C NMR chemical shifts of **1**–**19** showed systematically large deviations in proximity to the thienoquinoline moiety of the structures studied. These data suggest that the thienoquinoline structural component provides unpredictable stereoelectronic effects that introduce certain difficulties to the process of interpreting spectral data. Consequently, a number of carbon chemical shifts likely need to be reassigned pairwise. In addition, molecular analysis performed using the Natural Bond Orbitals approach demonstrated a systematic decrease in the *s*-character of the carbon–carbon *σ*-bonds in the inner turn of a helix.

## Figures and Tables

**Table 1:** Parameters of the orbitals mixing of *σ*_C=C_ bonds of internal and external turns of the helix of heterohelicenes **1**, **4**, **13**, and **15**.

Outer Helix			Inner Helix		
Bond	Fraction of *p ^a^*	*s*-Character	Bond	Fraction of *p ^a^*	*s*-Character
Compound: **1**					
C1-C2	1.73	0.37	C13-C13a	1.83	0.35
C2-C3	1.85	0.35	C13a-C13b	2.06	0.33
C3-C4	1.74	0.36	C13b-C13c	1.93	0.34
C4-C4a	1.90	0.34	C13c-C13d	1.95	0.34
C4a-C5	2.00	0.33	C13d-C1	1.90	0.35
C5-C6	1.67	0.38			
C10-C11	1.77	0.36			
C11-C12	1.82	0.35			
C12-C13	1.76	0.36			
Compound: **4**					
C1-C2	1.68	0.37	C11-C11a	1.83	0.35
C2-C2a	1.97	0.34	C11a-C11b	2.06	0.33
C2a-C3	1.97	0.34	C11b-C11c	1.91	0.34
C3-C4	1.69	0.37	C11c-C11d	1.98	0.34
C8-C9	1.77	0.36	C11d-C11e	2.01	0.33
C9-C10	1.82	0.36	C11e-C12	1.91	0.34
C13-C14	1.85	0.35			
C14-C15	1.74	0.37			
C15-C15a	1.92	0.34			
C15a-C1	1.99	0.33			
Compound: **13**					
C1-C2	3.47	0.37	C17-C17a	3.74	0.35
C2-C3	3.69	0.35	C17a-C17b	4.02	0.33
C3-C4	3.48	0.36	C17b-C17c	4.06	0.33
C4-C4a	3.80	0.34	C17c-C17d	3.85	0.34
C4a-C5	3.99	0.33	C17d-C17e	4.01	0.33
C5-C6	3.33	0.38	C17e-C1	3.82	0.34
C9b-C10	3.78	0.35			
C10-C11	3.50	0.36			
C11-C12	3.65	0.35			
C12-C13	3.55	0.36			
C13-C13a	3.79	0.35			
C13b-C14	3.77	0.35			
C14-C15	3.50	0.36			
C15-C16	3.67	0.35			
C16-C17	3.50	0.36			
Compound: **15**					
C1-C2	1.68	0.37	C13a-C13b	2.05	0.33
C2-C2a	1.97	0.34	C13b-C13c	1.92	0.34
C2a-C3	1.97	0.34	C13c-C13d	1.99	0.34
C3-C4	1.68	0.37	C13d-C13e	2.01	0.33
C7b-C8	1.93	0.34	C13e-C14	1.90	0.34
C8-C9	1.73	0.37			
C9-C10	1.86	0.35			
C10-C11	1.73	0.37			
C11-C11a	1.93	0.34			
C11a-C12	1.95	0.34			
C12-C13	1.68	0.37			
C15-C16	1.85	0.35			
C16-C17	1.74	0.37			
C17-C17a	1.91	0.34			
C17a-C1	1.98	0.34			

*^a^* The fraction of *s*-orbital is taken to be 1.

## Data Availability

Data are contained within the article.

## Supplementary Materials

The supporting information can be downloaded at: https://www.mdpi.com/article/10.3390/ijms25147733/s1. Cartesian coordinates, details of the computation of the ^1^H and ^13^C NMR chemical shifts, values of selected carbon bond lengths, optimized by the various DFT functionals are available at the “Supporting Information.pdf”. Complete data set of calculated chemical shifts are available at the “Supporting Information. Calculated ^1^H and ^13^C NMR chemical shifts of 1-19.xlsx”.
