# Supplementary material for: Computational NMR Study of Benzothienoquinoline Heterohelicenes
Source: Int J Mol Sci. 2024 Jul 15;25(14):7733. doi: 10.3390/ijms25147733 (PMC11277149; doi:10.3390/ijms25147733)
Supplement: Supplementary file 1 [file ijms-25-07733-s001.zip › ijms-3073443-supplementary.pdf]

SUPPORTING INFORMATION

Computational NMR Study of Benzothienoquinoline Heterohelicenes

Valentin A. Semenov,[1*] Gary E. Martin,[2*] and Leonid B. Krivdin[1]

[1] A.E. Favorsky Irkutsk Institute of Chemistry, Siberian Branch of the Russian Academy of Sciences, Favorsky St. 1, 664033 Irkutsk, Russia. E-mail: semenov@irioch.irk.ru
[2] Department of Chemistry and Biochemistry, Seton Hall University 400 South Orange Ave., South Orange, NJ 07079 USA. E-mail: gemartin.mrc@gmail.com

TABLE OF CONTENTS

Cartesian coordinates (Angstroms) of compounds 1-19, optimized at the M06-2X/cc-pVTZ//aug-cc-pVTZ level in the IEF-PCM media. 3
Compound: **1**. Solvent: Dimethylsulfoxide. 3
Compound: **2**. Solvent: Chloroform. 4
Compound: **3**. Solvent: Chloroform. 5
Compound: **4**. Solvent: Chloroform. 6
Compound: **5**. Solvent: Chloroform. 7
Compound: **6**. Solvent: Chloroform. 8
Compound: **7**. Solvent: Chloroform. 9
Compound: **8**. Solvent: Dimethylsulfoxide. 10
Compound: **9**. Solvent: Chloroform. 11
Compound: **10**. Solvent: Chloroform. 12
Compound: **11**. Solvent: Chloroform. 13
Compound: **12**. Solvent: Chloroform. 14
Compound: **13**. Solvent: Chloroform. 15
Compound: **14**. Solvent: Chloroform. 17
Compound: **15**. Solvent: Chloroform. 19
Compound: **16**. Solvent: Chloroform. 21
Compound: **17**. Solvent: Chloroform. 23
Compound: **18**. Solvent: Chloroform. 25
Compound: **19**. Solvent: Chloroform. 27
Details of ^{1}H and ^{13}C NMR calculations. 28
Values of selected carbon bond lengths of 4, optimized by the various DFT functionals. 30
Table S3. Calculated ^{1}H and ^{13}C NMR chemical shifts of benzothienoquinoline heterohelicenes 1-19. 31

Cartesian coordinates (Angstroms) of compounds 1-19, optimized at the M06-2X/cc-pVTZ//aug-cc-pVTZ level in the IEF-PCM media.

Compound: **1**. Solvent: Dimethylsulfoxide.

E^0: -1183.5830936 a.u.

C	-4.279243000	-1.453485000	0.153925000
C	-5.397397000	-0.643163000	0.530438000
C	-3.057303000	-0.840139000	-0.208935000
C	-2.921797000	0.597884000	-0.076460000
C	-4.100964000	1.355395000	0.094840000
C	-5.327778000	0.700710000	0.438694000
C	-1.702979000	1.335094000	-0.143111000
C	-1.811830000	2.708171000	-0.353784000
C	-3.056161000	3.353608000	-0.319834000
N	-4.156274000	2.708241000	-0.033227000
C	-0.305945000	0.956999000	0.053266000
C	0.558375000	2.044335000	-0.168698000
S	-0.286697000	3.524240000	-0.526556000
C	0.256446000	-0.227763000	0.552635000
C	1.620661000	-0.329153000	0.725172000
C	2.465770000	0.741210000	0.412408000
C	1.939993000	1.939732000	-0.022122000
C	-4.417132000	-2.851185000	0.047621000
C	-3.410257000	-3.620408000	-0.477747000
C	-2.242059000	-3.002323000	-0.948280000
C	-2.073921000	-1.645359000	-0.817965000
H	-6.318184000	-1.134098000	0.817666000
H	-6.184644000	1.331228000	0.631790000
H	-3.124413000	4.423917000	-0.477123000
H	-0.375143000	-1.058585000	0.825744000
H	2.040549000	-1.246159000	1.113917000
H	3.534390000	0.639814000	0.541101000
H	2.578094000	2.790053000	-0.218288000
H	-5.350330000	-3.303393000	0.358301000
H	-3.530395000	-4.691238000	-0.566676000
H	-1.475259000	-3.593135000	-1.430092000
H	-1.188721000	-1.182665000	-1.226360000

Compound: **2**. Solvent: Chloroform.

E^0: -1183.5913875 a.u.

C	-3.051157000	2.021476000	0.113427000
C	-4.270610000	1.281389000	0.053409000
C	-1.848613000	1.403431000	0.107712000
C	-1.739113000	-0.022932000	0.041868000
C	-2.927083000	-0.787352000	0.015941000
C	-4.215241000	-0.125419000	0.010832000
C	-0.501599000	-0.728778000	0.018987000
C	-0.569174000	-2.118079000	0.025647000
C	-1.801554000	-2.785896000	0.015663000
N	-2.938015000	-2.143289000	-0.000367000
C	0.885552000	-0.274294000	-0.008474000
C	1.779009000	-1.363517000	0.012629000
S	0.977288000	-2.907893000	0.038427000
C	1.442868000	1.011937000	-0.076076000
C	2.812560000	1.179152000	-0.094893000
C	3.673070000	0.079636000	-0.051617000
C	3.161159000	-1.199910000	-0.002872000
C	-5.522123000	1.928833000	0.047492000
C	-6.683153000	1.201204000	-0.000319000
C	-6.628313000	-0.202224000	-0.042080000
C	-5.419808000	-0.853129000	-0.035645000
H	-3.105447000	3.101184000	0.170482000
H	-0.960220000	2.004345000	0.177268000
H	-1.837123000	-3.869766000	0.014745000
H	0.824399000	1.890648000	-0.131282000
H	3.222659000	2.177784000	-0.148431000
H	4.743492000	0.230347000	-0.065131000
H	3.814315000	-2.061274000	0.017674000
H	-5.549292000	3.010616000	0.081538000
H	-7.640717000	1.703623000	-0.005290000
H	-7.546330000	-0.772755000	-0.079827000
H	-5.371942000	-1.931062000	-0.067575000

Compound: **3**. Solvent: Chloroform.

E^0: -1183.5790109 a.u.

C	-0.904060000	-1.315558000	0.771266000
C	0.256298000	-0.625568000	0.376230000
C	-2.139372000	-0.741945000	0.623439000
C	-2.283493000	0.552356000	0.074934000
C	-1.101398000	1.286241000	-0.210487000
C	0.155752000	0.655216000	-0.092619000
C	-3.536312000	1.235325000	-0.090328000
C	-3.457078000	2.611630000	-0.255031000
C	-2.227019000	3.257768000	-0.509174000
N	-1.097244000	2.616462000	-0.543269000
C	-4.917902000	0.796085000	-0.002171000
C	-5.777152000	1.886742000	0.109643000
S	-4.983616000	3.412520000	-0.103955000
C	-5.511279000	-0.510444000	-0.115528000
C	-6.896085000	-0.644528000	0.168936000
C	-7.689019000	0.507556000	0.441418000
C	-7.162421000	1.756106000	0.355403000
C	-7.502831000	-1.917334000	0.091265000
C	-6.795983000	-3.010879000	-0.329234000
C	-5.455172000	-2.858135000	-0.723006000
C	-4.831575000	-1.641755000	-0.619772000
H	-0.821021000	-2.301235000	1.208072000
H	1.224992000	-1.094726000	0.478688000
H	-3.013607000	-1.268960000	0.972154000
H	1.030388000	1.235422000	-0.352156000
H	-2.208087000	4.324685000	-0.704719000
H	-8.741033000	0.370885000	0.655544000
H	-7.776384000	2.636905000	0.481268000
H	-8.552340000	-2.003561000	0.342448000
H	-7.273335000	-3.978533000	-0.396722000
H	-4.913827000	-3.703252000	-1.125777000
H	-3.817095000	-1.537377000	-0.971268000

Compound: **4**. Solvent: Chloroform.

E^0: -1337.2701006 a.u.

C	0.570974000	-3.023477000	1.402984000
C	0.263196000	-1.732680000	1.029541000
C	1.243480000	-0.920017000	0.443896000
C	1.184510000	0.475923000	0.030498000
C	2.457940000	0.956730000	-0.254607000
S	3.696251000	-0.259540000	-0.225315000
C	2.542216000	-1.441770000	0.316793000
C	2.851624000	-2.753507000	0.668572000
C	1.854293000	-3.542120000	1.202447000
C	0.107694000	1.415503000	-0.005171000
C	0.491560000	2.778534000	0.038317000
N	1.758579000	3.213251000	-0.201945000
C	2.694999000	2.332067000	-0.416307000
C	-1.304163000	1.112536000	-0.016469000
C	-2.190221000	2.088733000	0.463347000
C	-1.735030000	3.418247000	0.698008000
C	-0.463570000	3.770845000	0.400487000
C	-1.873109000	-0.081876000	-0.602284000
C	-3.240029000	-0.376014000	-0.379744000
C	-4.060425000	0.560061000	0.317366000
C	-3.567272000	1.769552000	0.664769000
C	-1.157627000	-0.919911000	-1.485105000
C	-1.733912000	-2.037260000	-2.031395000
C	-3.059690000	-2.380472000	-1.720295000
C	-3.800005000	-1.552499000	-0.919501000
H	-0.189660000	-3.640986000	1.857610000
H	-0.729202000	-1.349039000	1.203853000
H	3.856033000	-3.132316000	0.547177000
H	2.074001000	-4.560098000	1.489945000
H	3.686883000	2.696335000	-0.655662000
H	-2.447706000	4.152889000	1.046959000
H	-0.117393000	4.791356000	0.473304000
H	-5.097818000	0.310347000	0.493865000
H	-4.205895000	2.522669000	1.105928000
H	-0.144935000	-0.666132000	-1.756257000
H	-1.164580000	-2.653430000	-2.712340000
H	-3.500660000	-3.272935000	-2.140171000
H	-4.839334000	-1.770496000	-0.713097000

Compound: **5**. Solvent: Chloroform.

E^0: -1337.221132 a.u.

C	-0.770579000	-2.087989000	0.075146000
C	-0.190431000	-3.333254000	0.032415000
C	1.203445000	-3.442645000	-0.008429000
C	1.989891000	-2.312765000	-0.004839000
C	1.422271000	-1.028380000	0.038759000
C	0.015712000	-0.924127000	0.078583000
C	2.223197000	0.193413000	0.045148000
C	1.555711000	1.451807000	0.084327000
C	0.118090000	1.500389000	0.123520000
C	-0.611716000	0.371619000	0.121753000
C	3.611199000	0.178625000	0.013873000
C	4.378267000	1.346684000	0.019052000
C	3.694432000	2.600923000	0.038539000
C	2.301906000	2.618294000	0.076493000
C	5.816154000	1.387385000	-0.006840000
C	6.401043000	2.638476000	-0.049165000
C	5.628789000	3.828247000	-0.036100000
N	4.335031000	3.818120000	0.014174000
C	6.822940000	0.336485000	0.000364000
C	8.123381000	0.877243000	-0.061287000
S	8.133344000	2.614577000	-0.107179000
C	6.710500000	-1.061293000	0.073546000
C	7.840425000	-1.852191000	0.066487000
C	9.116663000	-1.286768000	-0.010711000
C	9.266599000	0.082169000	-0.071331000
H	-1.847986000	-1.985765000	0.106410000
H	-0.805297000	-4.222628000	0.030002000
H	1.667816000	-4.418560000	-0.042948000
H	3.062648000	-2.433467000	-0.038084000
H	-0.357873000	2.471939000	0.154163000
H	-1.693100000	0.411959000	0.151546000
H	4.115058000	-0.767933000	-0.029465000
H	1.808196000	3.581187000	0.095293000
H	6.126252000	4.792053000	-0.063113000
H	5.752227000	-1.545507000	0.149734000
H	7.734727000	-2.926352000	0.124535000
H	9.989841000	-1.924076000	-0.016835000
H	10.247459000	0.534320000	-0.122077000

Compound: **6**. Solvent: Chloroform.

E^0: -1337.2266236 a.u.

C	0.187495000	-1.023345000	0.051946000
C	0.868261000	0.224728000	0.027155000
C	-1.167612000	-1.102869000	0.043613000
C	-1.982309000	0.062192000	0.010510000
C	-1.336091000	1.319926000	0.013151000
C	0.103336000	1.396383000	0.013843000
C	-3.409179000	0.049631000	-0.007885000
C	-4.047644000	1.282176000	0.021446000
C	-3.315835000	2.481407000	0.033614000
N	-2.012912000	2.499887000	0.020290000
C	-4.377677000	-1.040251000	-0.049241000
C	-5.697974000	-0.549196000	-0.019451000
S	-5.780581000	1.188088000	0.033898000
C	-4.210479000	-2.431200000	-0.131864000
C	-5.309444000	-3.265099000	-0.160370000
C	-6.606869000	-2.749035000	-0.111237000
C	-6.809613000	-1.386689000	-0.044952000
C	2.312704000	0.311225000	0.025241000
C	2.921790000	1.586513000	0.012575000
C	2.103002000	2.755683000	0.002269000
C	0.753418000	2.668004000	0.003205000
C	3.150380000	-0.824492000	0.034259000
C	4.516795000	-0.699063000	0.031807000
C	5.116037000	0.571022000	0.019964000
C	4.327748000	1.691356000	0.010277000
H	0.751456000	-1.942948000	0.083748000
H	-1.621658000	-2.076383000	0.082626000
H	-3.832070000	3.435115000	0.049453000
H	-3.232993000	-2.877398000	-0.188476000
H	-5.160931000	-4.333764000	-0.225168000
H	-7.455607000	-3.418271000	-0.132690000
H	-7.807685000	-0.971966000	-0.017409000
H	2.589589000	3.722931000	-0.006665000
H	0.138466000	3.554295000	-0.004309000
H	2.722911000	-1.815739000	0.042206000
H	5.136252000	-1.585436000	0.038564000
H	6.193641000	0.660312000	0.018056000
H	4.772966000	2.678242000	0.000680000

Compound: **7**. Solvent: Chloroform.

E^0: -1337.2154644 a.u.

C	-3.227554000	-4.111981000	0.497325000
C	-3.313334000	-2.758049000	0.839636000
C	-2.255103000	-1.903134000	0.616693000
C	-1.071656000	-2.382730000	0.034785000
C	-0.982264000	-3.763760000	-0.216910000
C	-2.057506000	-4.627270000	-0.018940000
C	0.194160000	-1.707475000	-0.231176000
C	1.190581000	-2.633290000	-0.520334000
S	0.617626000	-4.265795000	-0.683173000
C	0.592716000	-0.336428000	-0.156229000
C	1.981732000	-0.097977000	-0.058738000
N	2.933926000	-1.051248000	-0.265179000
C	2.546254000	-2.261514000	-0.556636000
C	-0.287343000	0.804682000	-0.211354000
C	0.189289000	2.073841000	0.153426000
C	1.577109000	2.234271000	0.433637000
C	2.443269000	1.205804000	0.270258000
C	-1.594415000	0.702027000	-0.782186000
C	-2.418944000	1.770964000	-0.859581000
C	-2.024116000	3.042172000	-0.348298000
C	-0.713578000	3.205170000	0.154215000
C	-2.910715000	4.137948000	-0.367562000
C	-2.522880000	5.362428000	0.108491000
C	-1.225431000	5.529047000	0.619852000
C	-0.342760000	4.478724000	0.637236000
H	-4.071955000	-4.765424000	0.667163000
H	-4.217911000	-2.377278000	1.292544000
H	-1.902699000	-0.239667000	-1.209340000
H	-1.962018000	-5.681235000	-0.239992000
H	3.311132000	-2.998688000	-0.773943000
H	1.963812000	3.205190000	0.702107000
H	3.509499000	1.339703000	0.388200000
H	-2.335947000	-0.868834000	0.913182000
H	-3.392720000	1.678539000	-1.323323000
H	-3.907232000	3.990934000	-0.764457000
H	-3.209681000	6.197428000	0.094501000
H	-0.919555000	6.491999000	1.005275000
H	0.642979000	4.638291000	1.047375000

Compound: **8**. Solvent: Dimethylsulfoxide.

E^0: -1337.224834 a.u.

C	1.278072000	-1.807962000	-0.021654000
C	2.622218000	-1.762613000	-0.031047000
C	0.494901000	-0.600038000	-0.015157000
C	1.151935000	0.664537000	-0.018745000
C	2.611156000	0.706265000	-0.029203000
C	3.329447000	-0.507610000	-0.035138000
C	-0.892246000	-0.668184000	-0.005302000
C	-1.693178000	0.474229000	0.001455000
C	-1.032126000	1.740968000	-0.002096000
C	0.362945000	1.802617000	-0.012015000
C	-3.131288000	0.484513000	0.011778000
C	-3.744295000	1.722886000	0.017276000
C	-2.996612000	2.928161000	0.013024000
N	-1.701046000	2.943887000	0.003786000
C	-4.112376000	-0.589317000	0.017870000
C	-5.426033000	-0.077794000	0.027703000
S	-5.476508000	1.659907000	0.029636000
C	-3.962603000	-1.985570000	0.015536000
C	-5.073770000	-2.802860000	0.022692000
C	-6.365126000	-2.266067000	0.032357000
C	-6.550260000	-0.899738000	0.034946000
C	4.734024000	-0.486070000	-0.045117000
C	5.421309000	0.704920000	-0.049264000
C	4.712706000	1.911453000	-0.043522000
C	3.335595000	1.909623000	-0.033699000
H	0.754985000	-2.755558000	-0.018697000
H	3.205313000	-2.674689000	-0.035807000
H	-1.335745000	-1.649019000	-0.003029000
H	0.795260000	2.792379000	-0.014133000
H	-3.513914000	3.881768000	0.017554000
H	-2.990505000	-2.447320000	0.008244000
H	-4.941431000	-3.875622000	0.020793000
H	-7.222590000	-2.924351000	0.037795000
H	-7.542720000	-0.470978000	0.042408000
H	5.267820000	-1.428018000	-0.049529000
H	6.502502000	0.710959000	-0.056932000
H	5.247343000	2.851322000	-0.046836000
H	2.815287000	2.856054000	-0.029510000

Compound: **9**. Solvent: Chloroform.

E^0: -1337.2167623 a.u.

C	-5.760445000	-1.891262000	0.198288000
C	-6.890294000	-1.116862000	0.611929000
C	-4.560767000	-1.239770000	-0.171916000
C	-4.459641000	0.197119000	-0.012121000
C	-5.652485000	0.922055000	0.195729000
C	-6.855517000	0.230014000	0.548484000
C	-3.260627000	0.966385000	-0.091620000
C	-3.410778000	2.339335000	-0.280681000
C	-4.669445000	2.952912000	-0.207700000
N	-5.745482000	2.275512000	0.092608000
C	-1.854715000	0.624065000	0.062812000
C	-1.029415000	1.718255000	-0.165980000
S	-1.907861000	3.184438000	-0.471733000
C	-1.257629000	-0.574359000	0.551195000
C	0.090731000	-0.664870000	0.688470000
C	0.950672000	0.420417000	0.352225000
C	0.387929000	1.647828000	-0.070566000
C	1.237559000	2.735561000	-0.363694000
C	2.595947000	2.605121000	-0.250957000
C	3.160608000	1.383738000	0.165034000
C	2.354200000	0.317472000	0.461612000
C	-5.864268000	-3.289347000	0.064512000
C	-4.847590000	-4.021479000	-0.493241000
C	-3.703413000	-3.364376000	-0.969460000
C	-3.567491000	-2.006859000	-0.813576000
H	-7.793632000	-1.636282000	0.904706000
H	-7.722630000	0.836474000	0.769995000
H	-4.768979000	4.023598000	-0.346063000
H	-1.881720000	-1.403670000	0.844771000
H	0.536048000	-1.573498000	1.072087000
H	0.808039000	3.675199000	-0.687888000
H	3.239830000	3.442033000	-0.482966000
H	4.234543000	1.292652000	0.251153000
H	2.781785000	-0.622411000	0.786628000
H	-6.780371000	-3.771819000	0.380666000
H	-4.942600000	-5.092905000	-0.603204000
H	-2.930580000	-3.925389000	-1.476661000
H	-2.701995000	-1.512531000	-1.227762000

Compound: **10**. Solvent: Chloroform.

E^0: -1337.2168803 a.u.

C	0.197156000	-1.297698000	0.778305000
C	1.383428000	-0.601157000	0.390507000
C	-1.023042000	-0.729503000	0.655158000
C	-1.179248000	0.599184000	0.142370000
C	-0.020876000	1.364483000	-0.107154000
C	1.282644000	0.737043000	-0.039380000
C	-2.441523000	1.248990000	-0.002114000
C	-2.407442000	2.637950000	-0.112259000
C	-1.199382000	3.318496000	-0.320583000
N	-0.052021000	2.695221000	-0.372491000
C	-3.810865000	0.762800000	0.055795000
C	-4.704879000	1.818687000	0.209201000
S	-3.962406000	3.380603000	0.067480000
C	-4.359599000	-0.554304000	-0.128255000
C	-5.742002000	-0.747009000	0.134089000
C	-6.573510000	0.364021000	0.454546000
C	-6.086886000	1.631823000	0.434152000
C	2.650882000	-1.211433000	0.465629000
C	3.781551000	-0.517573000	0.117752000
C	3.680664000	0.816747000	-0.309902000
C	2.456563000	1.434458000	-0.381156000
C	-6.306557000	-2.033183000	-0.013163000
C	-5.560851000	-3.080942000	-0.479829000
C	-4.221872000	-2.865801000	-0.851189000
C	-3.638870000	-1.636868000	-0.681238000
H	0.294564000	-2.293557000	1.191549000
H	-1.896969000	-1.261509000	0.996918000
H	-1.193815000	4.393987000	-0.459300000
H	-7.622380000	0.183864000	0.651260000
H	-6.731070000	2.484848000	0.595057000
H	2.715394000	-2.237993000	0.803494000
H	4.750944000	-0.993530000	0.174532000
H	4.574689000	1.360598000	-0.583098000
H	2.372567000	2.461292000	-0.704229000
H	-7.354904000	-2.165228000	0.222408000
H	-6.005855000	-4.058748000	-0.600706000
H	-3.649838000	-3.672129000	-1.289475000
H	-2.624443000	-1.483515000	-1.014910000

Compound: **11**. Solvent: Chloroform.

E^0: -1337.2193024 a.u.

C	-1.972712000	-1.547222000	-0.116816000
C	-3.166547000	-0.764599000	0.196096000
C	-0.763734000	-0.887205000	-0.413549000
C	-0.685096000	0.568227000	-0.320699000
C	-1.884933000	1.296346000	-0.330061000
C	-3.143924000	0.623670000	-0.016088000
C	0.517400000	1.324047000	-0.284788000
C	0.416667000	2.684447000	-0.579947000
C	-0.830361000	3.289338000	-0.751303000
N	-1.944032000	2.625726000	-0.560860000
C	1.887661000	0.981984000	0.091488000
C	2.755878000	2.075444000	-0.073177000
S	1.941509000	3.520822000	-0.604897000
C	2.403392000	-0.173054000	0.697233000
C	3.738346000	-0.241517000	1.035800000
C	4.597726000	0.833556000	0.787028000
C	4.110332000	2.003731000	0.243974000
C	-2.023199000	-2.945155000	-0.235150000
C	-0.941425000	-3.667647000	-0.685066000
C	0.221921000	-3.002593000	-1.074149000
C	0.299384000	-1.635054000	-0.944638000
C	-4.317985000	1.371398000	0.156005000
C	-5.484732000	0.765763000	0.562394000
C	-5.502491000	-0.607964000	0.814821000
C	-4.362614000	-1.357663000	0.630324000
H	-0.907658000	4.342545000	-0.995570000
H	1.755555000	-1.006948000	0.918852000
H	4.122347000	-1.136144000	1.505419000
H	5.644402000	0.758645000	1.047330000
H	4.755201000	2.857810000	0.090539000
H	-2.939597000	-3.470111000	-0.011861000
H	-1.011675000	-4.742618000	-0.777841000
H	1.052319000	-3.553452000	-1.493639000
H	1.183841000	-1.121157000	-1.289289000
H	-4.279641000	2.435538000	-0.020580000
H	-6.381329000	1.354498000	0.699630000
H	-6.411128000	-1.085994000	1.154325000
H	-4.398712000	-2.417497000	0.833268000

Compound: **12**. Solvent: Chloroform.

E^0: -1337.2164596 a.u.

C	2.598340000	-0.026921000	1.017577000
C	3.456884000	1.034175000	0.677678000
C	1.259344000	0.043731000	0.736748000
C	0.704189000	1.178365000	0.103120000
C	1.556738000	2.289516000	-0.133000000
C	2.939817000	2.173144000	0.124861000
C	-0.690127000	1.345298000	-0.201794000
C	-1.102337000	2.646844000	-0.452868000
C	-0.176546000	3.694351000	-0.650949000
N	1.106709000	3.516156000	-0.550048000
C	-1.820943000	0.436549000	-0.186660000
C	-3.015332000	1.125391000	-0.211855000
S	-2.821269000	2.827188000	-0.473218000
C	-1.871712000	-1.012442000	-0.274547000
C	-3.102749000	-1.672596000	-0.053237000
C	-4.322178000	-0.899309000	0.160809000
C	-4.291359000	0.499264000	-0.011205000
C	-5.476757000	1.247708000	0.079534000
C	-6.672792000	0.634642000	0.360805000
C	-6.707746000	-0.748424000	0.568526000
C	-5.557275000	-1.495802000	0.468242000
C	-3.136531000	-3.075013000	-0.145449000
C	-2.024595000	-3.800363000	-0.502463000
C	-0.838610000	-3.136498000	-0.824315000
C	-0.771064000	-1.768012000	-0.715793000
H	2.994553000	-0.900815000	1.516116000
H	4.515263000	0.960748000	0.886110000
H	0.610633000	-0.763046000	1.040386000
H	3.561963000	3.028683000	-0.099029000
H	-0.530322000	4.685380000	-0.914421000
H	-5.440442000	2.321121000	-0.056696000
H	-7.578997000	1.219229000	0.434633000
H	-7.643152000	-1.234417000	0.808883000
H	-5.616950000	-2.560242000	0.636659000
H	-4.061680000	-3.602582000	0.029497000
H	-2.083236000	-4.877474000	-0.574702000
H	0.020065000	-3.692428000	-1.174615000
H	0.132834000	-1.258370000	-1.010856000

Compound: **13**. Solvent: Chloroform.

E^0: -1490.8477048 a.u.

C	-4.978967000	-0.797143000	3.281405000
C	-5.741344000	-1.989949000	3.073653000
C	-3.653886000	-0.717674000	2.793990000
C	-3.122196000	-1.810787000	2.005779000
C	-3.824784000	-3.035519000	2.025984000
C	-5.168247000	-3.078324000	2.520467000
C	-1.882420000	-1.803560000	1.292415000
C	-1.319701000	-3.057792000	1.044713000
C	-2.054898000	-4.238194000	1.215314000
N	-3.299929000	-4.220600000	1.612784000
C	-1.022189000	-0.751860000	0.771536000
C	0.212041000	-1.246071000	0.407229000
S	0.307474000	-2.975147000	0.460742000
C	-1.331692000	0.621191000	0.414100000
C	-0.285972000	1.482623000	0.010465000
C	1.076712000	0.974382000	-0.111436000
C	1.308458000	-0.410668000	0.006551000
C	2.593764000	-0.932995000	-0.213241000
C	3.644155000	-0.102903000	-0.519131000
C	3.431378000	1.277167000	-0.603616000
C	2.174697000	1.800888000	-0.405348000
C	-0.606779000	2.802384000	-0.351577000
C	-1.907531000	3.248294000	-0.364407000
C	-2.945138000	2.370612000	-0.042789000
C	-2.657357000	1.081546000	0.337613000
C	-2.866196000	0.379563000	3.196680000
C	-3.390736000	1.388117000	3.966459000
C	-4.734211000	1.349587000	4.367468000
C	-5.508094000	0.266073000	4.038158000
H	-6.761408000	-2.021423000	3.434228000
H	-5.691863000	-4.020127000	2.433494000
H	-1.614395000	-5.199091000	0.974390000
H	2.758177000	-1.999031000	-0.120451000
H	4.631549000	-0.512269000	-0.679861000
H	4.258110000	1.937666000	-0.825561000
H	2.043467000	2.870131000	-0.473004000
H	0.174177000	3.479034000	-0.663565000
H	-2.125839000	4.265860000	-0.657394000
H	-3.974110000	2.697415000	-0.101757000
H	-3.465549000	0.401083000	0.555623000
H	-1.823798000	0.415433000	2.918951000
H	-2.760631000	2.213074000	4.269151000

H	-5.146096000	2.154934000	4.959865000
H	-6.532278000	0.195994000	4.381927000

Compound: **14**. Solvent: Chloroform.

E^0: -1490.8542921 a.u.

C	2.706317000	-0.011062000	0.867542000
C	3.465759000	1.150384000	0.525469000
C	1.376073000	-0.073559000	0.636794000
C	0.668186000	1.025630000	0.050347000
C	1.360946000	2.234982000	-0.166413000
C	2.796420000	2.283166000	0.020970000
C	-0.736244000	1.017375000	-0.204772000
C	-1.333014000	2.262180000	-0.393805000
C	-0.560726000	3.419248000	-0.566192000
N	0.744466000	3.395527000	-0.507931000
C	-1.734018000	-0.038410000	-0.193581000
C	-3.009585000	0.480623000	-0.150416000
S	-3.062196000	2.203665000	-0.346982000
C	-1.590096000	-1.474433000	-0.352540000
C	-2.710877000	-2.307331000	-0.127592000
C	-4.014085000	-1.719061000	0.164717000
C	-4.179754000	-0.323026000	0.060615000
C	-5.451084000	0.251250000	0.227003000
C	-6.540994000	-0.531719000	0.517420000
C	-6.379976000	-1.914355000	0.658449000
C	-5.143890000	-2.492102000	0.483703000
C	-2.558022000	-3.695160000	-0.291791000
C	-1.372164000	-4.243170000	-0.720004000
C	-0.299560000	-3.408265000	-1.042356000
C	-0.413890000	-2.050138000	-0.865251000
C	4.860586000	1.197845000	0.717388000
C	5.569311000	2.331516000	0.411883000
C	4.901546000	3.460839000	-0.090199000
C	3.541438000	3.439736000	-0.276633000
H	3.217597000	-0.843943000	1.333241000
H	0.821254000	-0.946731000	0.942568000
H	-1.038329000	4.372059000	-0.765939000
H	-5.565175000	1.324529000	0.142994000
H	-7.514379000	-0.080810000	0.650047000
H	-7.230719000	-2.534001000	0.905943000
H	-5.051927000	-3.560973000	0.602375000
H	-3.396413000	-4.352031000	-0.117141000
H	-1.287039000	-5.313423000	-0.847021000
H	0.612956000	-3.824011000	-1.446718000
H	0.401866000	-1.408348000	-1.160223000
H	5.362183000	0.322883000	1.111041000
H	6.640297000	2.359802000	0.559060000

H	5.463875000	4.353012000	-0.329945000
H	3.021202000	4.305615000	-0.657765000

Compound: **15**. Solvent: Chloroform.

E^0: -1490.846512 a.u.

C	-6.574430000	0.575564000	-1.625621000
C	-7.531633000	1.378634000	-0.941325000
C	-5.250608000	1.019577000	-1.765584000
C	-4.832034000	2.157161000	-0.981808000
C	-5.834552000	3.027478000	-0.487811000
C	-7.193991000	2.603859000	-0.476590000
C	-3.494704000	2.481710000	-0.590513000
C	-3.270607000	3.765278000	-0.103100000
C	-4.336028000	4.644685000	0.150306000
N	-5.577290000	4.260144000	0.033671000
C	-2.290395000	1.676177000	-0.486028000
C	-1.202627000	2.425702000	-0.058878000
S	-1.610958000	4.076805000	0.298776000
C	-2.127643000	0.269641000	-0.634875000
C	-0.913009000	-0.303671000	-0.430328000
C	0.228330000	0.470901000	-0.073496000
C	0.090050000	1.864839000	0.129898000
C	1.214939000	2.624890000	0.515030000
C	2.433866000	2.024538000	0.683130000
C	2.577393000	0.638283000	0.478067000
C	1.497835000	-0.119244000	0.110047000
C	-4.429963000	0.382361000	-2.773280000
C	-4.856529000	-0.845509000	-3.335028000
C	-6.135288000	-1.375594000	-2.988639000
C	-6.982531000	-0.658654000	-2.216717000
C	-4.049975000	-1.494459000	-4.293094000
C	-2.893044000	-0.917606000	-4.745944000
C	-2.521594000	0.351290000	-4.271909000
C	-3.269465000	0.981413000	-3.310762000
H	-8.549322000	1.017798000	-0.868386000
H	-7.920951000	3.275817000	-0.042510000
H	-4.149041000	5.647120000	0.518323000
H	-2.977001000	-0.345804000	-0.887051000
H	-0.795173000	-1.374406000	-0.534024000
H	1.109390000	3.691327000	0.670514000
H	3.291353000	2.615231000	0.974105000
H	3.544681000	0.174702000	0.614285000
H	1.601120000	-1.185621000	-0.045030000
H	-6.437281000	-2.323412000	-3.415288000
H	-7.989676000	-1.006764000	-2.026997000
H	-4.382242000	-2.446419000	-4.687465000
H	-2.287549000	-1.416865000	-5.489726000

H	-1.643408000	0.839455000	-4.671581000
H	-2.976002000	1.965856000	-2.979565000

Compound: **16**. Solvent: Chloroform.

E^0: -1490.8515905 a.u.

C	-4.173661000	-0.597974000	-0.115723000
C	-4.705319000	-1.875850000	-0.452717000
C	-2.817430000	-0.498305000	0.233907000
C	-1.957105000	-1.647785000	0.107031000
C	-2.560486000	-2.915409000	-0.046490000
C	-3.945039000	-2.993240000	-0.358732000
C	-0.527593000	-1.635249000	0.165635000
C	0.097876000	-2.858004000	0.390210000
C	-0.622883000	-4.064867000	0.372233000
N	-1.895759000	-4.098413000	0.092227000
C	0.458759000	-0.589762000	-0.053062000
C	1.752870000	-1.047779000	0.164482000
S	1.821634000	-2.741665000	0.536979000
C	0.292481000	0.721305000	-0.586936000
C	1.369225000	1.526663000	-0.779326000
C	2.690044000	1.098354000	-0.458632000
C	2.899239000	-0.220379000	0.009575000
C	4.211752000	-0.657850000	0.286668000
C	5.275588000	0.186948000	0.114919000
C	5.071584000	1.502092000	-0.345960000
C	3.806830000	1.944984000	-0.627808000
C	-2.357416000	0.703482000	0.858018000
C	-3.163060000	1.780233000	1.000128000
C	-4.501229000	1.766938000	0.507502000
C	-5.019261000	0.574532000	-0.046230000
C	-5.316775000	2.913487000	0.594254000
C	-6.607157000	2.892868000	0.134971000
C	-7.125137000	1.714415000	-0.426912000
C	-6.352440000	0.583730000	-0.510146000
H	-5.748661000	-1.974375000	-0.709635000
H	-4.362934000	-3.977076000	-0.520524000
H	-0.116154000	-5.009305000	0.536384000
H	-0.690126000	1.065357000	-0.869697000
H	1.237325000	2.516150000	-1.197208000
H	4.372749000	-1.666882000	0.645122000
H	6.277488000	-0.154930000	0.334407000
H	5.919279000	2.160103000	-0.478158000
H	3.644102000	2.953210000	-0.986824000
H	-1.361252000	0.725971000	1.272593000
H	-2.804104000	2.669350000	1.502560000
H	-4.900707000	3.813352000	1.029391000
H	-7.225436000	3.777480000	0.200727000

H	-8.140346000	1.697826000	-0.798985000
H	-6.777799000	-0.301718000	-0.957953000

Compound: **17**. Solvent: Chloroform.

E^0: -1490.8554127 a.u.

C	-4.251889000	-1.892260000	-0.122360000
C	-5.441718000	-1.131858000	0.253667000
C	-3.064497000	-1.209644000	-0.453608000
C	-3.002620000	0.244124000	-0.337188000
C	-4.210004000	0.956287000	-0.284586000
C	-5.445710000	0.260730000	0.068910000
C	-1.808631000	1.015913000	-0.341565000
C	-1.940881000	2.378505000	-0.616968000
C	-3.201289000	2.970022000	-0.723625000
N	-4.296585000	2.288932000	-0.492913000
C	-0.422786000	0.689690000	-0.035562000
C	0.417621000	1.778469000	-0.227301000
S	-0.426579000	3.223492000	-0.695315000
C	0.125554000	-0.487293000	0.551148000
C	1.453476000	-0.563208000	0.827219000
C	2.338276000	0.516933000	0.543396000
C	1.817444000	1.722923000	0.017510000
C	2.688331000	2.805911000	-0.227264000
C	4.027922000	2.691442000	0.032002000
C	4.551438000	1.491215000	0.550842000
C	3.723346000	0.429959000	0.801905000
C	-6.621001000	0.988901000	0.305069000
C	-7.762005000	0.359567000	0.746951000
C	-7.751855000	-1.019124000	0.971364000
C	-6.611229000	-1.749492000	0.724588000
C	-4.288114000	-3.288226000	-0.265174000
C	-3.214584000	-3.986861000	-0.769502000
C	-2.075911000	-3.298487000	-1.189834000
C	-2.012108000	-1.932423000	-1.038617000
H	-3.302531000	4.025233000	-0.949909000
H	-0.521804000	-1.312654000	0.803245000
H	1.861685000	-1.455647000	1.283227000
H	2.290801000	3.729253000	-0.629732000
H	4.688616000	3.524778000	-0.162619000
H	5.610894000	1.412505000	0.751906000
H	4.119103000	-0.493602000	1.204709000
H	-6.603631000	2.056823000	0.149005000
H	-8.659445000	0.933345000	0.933239000
H	-8.639540000	-1.516096000	1.338061000
H	-6.625376000	-2.813554000	0.907327000
H	-5.188179000	-3.829928000	-0.016861000
H	-3.273817000	-5.060845000	-0.879751000

H	-1.254726000	-3.829732000	-1.650662000
H	-1.148894000	-1.399871000	-1.408435000

Compound: **18**. Solvent: Chloroform.

E^0: -1644.4864016 a.u.

C	1.252474000	0.086774000	3.923982000
C	2.373070000	1.024067000	3.941658000
C	0.044620000	0.456742000	3.300537000
C	-0.064990000	1.746006000	2.625062000
C	0.878102000	2.736675000	2.941603000
C	2.153291000	2.352879000	3.543849000
C	-1.135128000	2.134081000	1.767575000
C	-1.360187000	3.510736000	1.657400000
C	-0.434512000	4.435170000	2.141909000
N	0.691878000	4.051443000	2.692121000
C	-2.108428000	1.397180000	0.973843000
C	-3.120331000	2.219048000	0.528772000
S	-2.843060000	3.900075000	0.851463000
C	-2.061875000	0.040083000	0.460827000
C	-3.190986000	-0.483979000	-0.209552000
C	-4.359129000	0.358273000	-0.446628000
C	-4.289083000	1.733974000	-0.148671000
C	1.316106000	-1.146590000	4.591014000
C	0.217634000	-1.971731000	4.680397000
C	-0.998583000	-1.569253000	4.127636000
C	-1.078067000	-0.373153000	3.453024000
C	-3.124052000	-1.795773000	-0.710158000
C	-1.978805000	-2.549051000	-0.601740000
C	-0.839070000	-1.998040000	-0.011919000
C	-0.884571000	-0.726251000	0.507219000
C	-5.364331000	2.581068000	-0.464252000
C	-6.504652000	2.079703000	-1.042058000
C	-6.597184000	0.709870000	-1.310714000
C	-5.546149000	-0.128999000	-1.020118000
C	3.648781000	0.664383000	4.404270000
C	4.659891000	1.594204000	4.497646000
C	4.424390000	2.922816000	4.136135000
C	3.186448000	3.293772000	3.663608000
H	-0.607609000	5.500129000	2.037077000
H	2.230635000	-1.444834000	5.081036000
H	0.291127000	-2.911742000	5.209703000
H	-1.879559000	-2.186337000	4.236765000
H	-2.029179000	-0.054586000	3.054041000
H	-3.973749000	-2.218954000	-1.223886000
H	-1.953038000	-3.552075000	-1.004537000
H	0.081610000	-2.563475000	0.029077000
H	0.008638000	-0.297429000	0.933512000

H	-5.297045000	3.636163000	-0.231228000
H	-7.330208000	2.737227000	-1.275426000
H	-7.499265000	0.306018000	-1.749071000
H	-5.649916000	-1.181399000	-1.236189000
H	3.853832000	-0.357848000	4.685238000
H	5.636072000	1.292773000	4.852156000
H	5.215927000	3.655324000	4.214947000
H	2.992503000	4.311436000	3.360438000

Compound: **19**. Solvent: Chloroform.

E^0: -1353.2543852 a.u.

C	8.369956000	8.288322000	2.919259000
H	7.743450000	9.042361000	3.371754000
C	9.655605000	8.110297000	3.356510000
H	10.069410000	8.725964000	4.141685000
C	10.433205000	7.103140000	2.767119000
H	11.465099000	6.966906000	3.070065000
N	9.970018000	6.267395000	1.865036000
C	8.687242000	6.410324000	1.459079000
C	8.155247000	5.398833000	0.598977000
H	8.826787000	4.621544000	0.262474000
C	6.838786000	5.384695000	0.307075000
H	6.389756000	4.585124000	-0.265674000
C	5.964565000	6.427200000	0.755939000
N	4.637688000	6.236292000	0.528882000
C	3.799869000	7.125263000	0.993449000
H	2.739817000	6.922404000	0.890631000
C	4.239138000	8.332335000	1.555038000
S	3.203043000	9.591037000	2.140221000
C	4.573760000	10.624166000	2.380399000
C	4.475273000	11.945966000	2.870522000
H	3.523927000	12.327522000	3.213698000
C	5.595871000	12.712492000	2.894149000
H	5.559333000	13.723079000	3.279582000
C	6.819729000	12.241499000	2.336752000
C	7.924017000	13.112238000	2.212379000
H	7.844230000	14.108592000	2.628190000
C	9.056968000	12.722382000	1.550459000
H	9.892632000	13.400890000	1.450260000
C	9.112697000	11.446685000	0.963246000
H	9.982195000	11.156595000	0.389417000
C	8.069008000	10.568238000	1.101012000
H	8.120003000	9.605517000	0.616646000
C	6.911593000	10.919370000	1.828949000
C	5.778746000	10.047549000	1.987626000
C	5.597859000	8.645118000	1.641445000
C	6.497152000	7.557054000	1.413062000
C	7.855125000	7.462234000	1.902976000

Details of ^{1}H and ^{13}C NMR calculations.

All calculations of ^{1}H and ^{13}C NMR isotropic magnetic shielding constants (the latter being converted into chemical shifts) of **1-19** were carried out at the DFT level in the liquid phase by applying the Gaussian 09.[21] In these calculations, we used the functional of Perdew, Burke, and Ernzerhof[24,25] with a predetermined amount of the exact exchange, the latter known as PBE0.[26] This functional was used here within the recently proposed pecS-2 basis set, which was specially developed for calculating shielding constants in large synthetic compounds and natural products.[27,28] This basis set makes it possible to achieve better correlation with experimental data (and, accordingly, higher accuracy) at an acceptable computational resource cost.

Calculated proton and carbon isotropic magnetic shielding constants of **1-19** were then converted into ^{1}H and ^{13}C NMR chemical shifts, as recommended by the International Union of Pure and Applied Chemistry (IUPAC): [29]

$$\delta_{calc} = \frac{\sigma_{TMS} - \sigma_{calc}}{1 - 10^{-6}\sigma_{TMS}}$$

To account for systematic errors of calculated chemical shifts, we have established correlations between their isotropic magnetic shielding constants (*y*) and experimental chemical shifts (*x*). Resulting linear regressions were further used to define the equations of the y = *a*x + *b* type. The slope *a* and intercept *b* were then used for recalculating the unscaled shielding constants (σ_{calc}) into the scaled values of chemical shifts (δ_s) as $\delta_s = (\sigma_{calc} - b)/a$. The slope and intercept parameters of compounds **1-19** are provided in the Table S1:

Table S1. Linear regression parameters of correlation plots of **1-19**.

Cmpd	^{1}H NMR		^{13}C NMR	
	slope	intercept	slope	intercept
**1**	1.0369	31.28	1.0975	187.04
**2**	0.8653	29.60	-	-
**3**	1.0194	31.08	1.0900	186.83
**4**	1.0036	31.03	1.0819	186.41
**5**	1.1700	32.24	1.0883	186.16
**6**	1.1275	31.92	1.0906	186.56
**7**	1.0847	31.58	1.0912	186.60
**8**	-	-	-	-
**9**	1.0646	31.42	1.0826	185.52

**10**	1.0776	31.54	1.0892	186.44
**11**	1.1166	31.89	1.1053	188.40
**12**	1.0947	31.64	1.1023	188.03
**13**	1.0646	31.42	1.1041	188.27
**14**	1.0938	31.61	1.0925	186.81
**15**	1.0314	31.21	1.0691	183.80
**16**	1.0102	31.12	0.9883	173.37
**17**	1.1113	31.74	1.1039	188.18
**18**	1.1102	31.82	1.1182	190.23
**19**	0.9971	30.97	1.0781	185.19

Values of selected carbon bond lengths of 4, optimized by the various DFT functionals.

Table S2. Values of selected carbon bond lengths (Å) of [1]benzothieno[2,3-*c*]naphtho[1,2-*f*]quinoline (**4**), optimized by the various DFT functionals in combination with cc-pVTZ//aug-cc-pVTZ LDBS basis set scheme. The solvent effect was accounted for within the IEF-PCM.

**Method**	**Inner bonds**						**Outer bonds**			
	$11\text{-}11_a$	$11_a\text{-}11_b$	$11_b\text{-}11_c$	$11_c\text{-}11_d$	$11_d\text{-}11_e$	$11_e\text{-}12$	8-9	3-4	1-2	14-15
					GGA					
OLYP	1.410	1.458	1.439	1.451	1.454	1.418	1.390	1.366	1.365	1.381
					meta-GGA					
M06-L	1.401	1.446	1.427	1.439	1.441	1.409	1.381	1.357	1.357	1.372
TPSS	1.409	1.457	1.437	1.449	1.451	1.417	1.390	1.365	1.364	1.380
					hybrid GGA					
B3LYP	1.404	1.458	1.433	1.448	1.450	1.413	1.384	1.357	1.356	1.373
B3PW91	1.402	1.453	1.430	1.443	1.446	1.411	1.382	1.356	1.356	1.372
BHandHLYP	1.394	1.454	1.425	1.442	1.443	1.406	1.373	1.345	1.344	1.362
mPW1PW91	1.400	1.452	1.428	1.442	1.444	1.409	1.380	1.354	1.353	1.370
PBE0	1.400	1.452	1.429	1.442	1.444	1.410	1.380	1.354	1.354	1.371
					hybrid meta-GGA					
M06-2X	1.402	1.457	1.430	1.444	1.447	1.412	1.379	1.353	1.352	1.369
					with dispersion model (range-separated hybrid)					
B97D	1.409	1.453	1.434	1.447	1.450	1.417	1.392	1.368	1.367	1.382
ωB97XD	1.399	1.457	1.428	1.444	1.447	1.411	1.379	1.351	1.350	1.368

Table S3. Calculated ^{1}H and ^{13}C NMR chemical shifts of benzothienoquinoline heterohelicenes 1-19.

Nuclei	1		2		3		4		5		6		7		8		9		10	
	^{1}H NMR	^{13}C NMR	^{1}H NMR	^{13}C NMR	^{1}H NMR	^{13}C NMR	^{1}H NMR	^{13}C NMR	^{1}H NMR	^{13}C NMR	^{1}H NMR	^{13}C NMR	^{1}H NMR	^{13}C NMR	^{1}H NMR	^{13}C NMR	^{1}H NMR	^{13}C NMR	^{1}H NMR	^{13}C NMR
1	9.31	128.1	8.63	139.5	9.03	125.9		129.02	9.05	122.9	8.88	123.0	8.01	128.6	8.67	141.2	9.13	128.7	7.98	127.7
2	7.64	124.3	8.30	139.1	7.56	125.0		126.21	7.79	126.7	7.74	126.3	7.65	126.6	8.34	139.4	7.54	124.3	7.69	127.2
2a							8.08	131.39												
3	7.77	127.4	8.37	138.8	7.70	127.8		128.61	7.68	127.0	7.67	126.4	7.73	126.5	8.45	139.1	7.64	127.2	7.75	126.8
4	8.14	127.9	10.32	137.2	8.27	131.1		129.96	7.93	129.1	8.05	129.0	8.91	123.7	9.80	134.8	8.01	128.0	9.55	125.5
4a		131.8		144.6		145.3	7.91	144.87		132.0		132.3		129.8		142.0		132.2		132.0
4b				155.3										129.3		141.5				142.3
5	8.13	129.2							7.79	128.3	8.09	128.3	8.98	123.6	9.50	136.8	8.00	129.2		
5a																156.1				
6	8.08	128.2	9.97	156.2	9.20	144.8	6.99	144.19	7.94	127.6	9.57	123.7	8.28	129.2			8.04	129.1	9.27	142.6
6a		143.7		148.8		135.2	9.37	135.01		130.9		130.8		143.7				144.5		135.6
6b												141.9								
7									8.74	130.4					9.96	159.8				
7a				157.2		143.6	8.11	142.20		142.6						147.9				143.7
7b																				
8	9.22	143.8	8.67	135.6	7.93	121.3	8.28	122.62			9.23	143.4	9.12	144.2			9.18	143.9	7.96	121.0
8a		136.1										135.9		136.4		157.0		135.9		
8b																				
9			8.22	139.2	8.03	130.5	8.11	127.28	9.07	145.8					8.81	136.0			8.05	130.6
9a		143.3				131.9				135.0		143.8		143.5				143.3		131.5
9b																		128.6		
10	8.11	123.6	8.24	136.4	8.05	129.4	6.74	122.22			8.04	124.0	7.98	123.5	8.34	139.3	8.36	124.8	8.06	129.1
10a										143.6										
11	7.66	128.2	9.69	138.5	7.57	125.8	6.95	128.19	8.06	124.1	7.64	127.3	7.54	127.8	8.42	137.2	7.63	126.9	7.60	125.4
11a				147.7			7.94	134.08												
11b				147.6			7.34	136.41												
11c				134.2			8.02	119.01												
11d							7.48	125.20												
11e								129.57												
12	7.47	122.8	9.70	132.4	7.60	125.7		129.25	7.67	126.8	7.67	124.8	7.38	123.0	9.95	138.4	7.61	127.4	7.64	125.7
12a																147.6				
12b																145.9				
12c																135.2				
13	9.04	126.4	8.75	141.1	9.10	126.3		125.93	7.78	125.0	8.99	126.7	8.80	127.3	9.89	134.4	7.97	129.1	8.97	126.3
13a		134.3		144.1		130.4						135.1		135.0		143.2		132.2		130.1
13b		135.5				130.2						134.8		135.4						129.6
13c		122.2				136.9						123.0		122.5						137.4
13d		127.7				124.5								126.4						121.6
13e																				
14								125.82	9.20	126.4	9.15	121.3	8.93	127.0	8.80	139.9	7.74	124.2	8.78	124.3
14a										135.1										
14b										133.6										
14c										123.6										
15								128.24	10.25	116.8	9.06	123.0	7.83	125.0	8.55	141.3	8.74	124.1	7.91	126.1
15a						131.03				129.1		128.5		132.2		144.0		132.5		132.4
15b										130.1		129.5						136.6		
15c																		122.2		
15d																		128.2		
15e																				
16																				
17																				
17a																				
17b																				
17c																				
17d																				
17e																				
18																				
19																				
19a																				
19b																				
19c																				
19d																				
19e																				

Nuclei	11		12		13		14		15		16		17		18		19	
	^{1}H NMR	^{13}C NMR	^{1}H NMR	^{13}C NMR	^{1}H NMR	^{13}C NMR	^{1}H NMR	^{13}C NMR	^{1}H NMR	^{13}C NMR	^{1}H NMR	^{13}C NMR	^{1}H NMR	^{13}C NMR	^{1}H NMR	^{13}C NMR	^{1}H NMR	^{13}C NMR
1	9.00	129.3	8.85	125.7	7.88	130.4	7.92	127.7	8.12	129.0	8.17	128.6	8.87	129.6	7.80	130.8	8.23	136.7
2	7.50	124.6	7.51	124.6	7.00	124.7	7.63	127.2	8.04	126.2	7.78	126.4	7.44	124.7	6.98	125.4	6.89	118.7
2a										131.4								
3	7.72	128.1	7.68	127.6	7.49	126.5	7.70	126.7	8.14	128.7	7.86	126.4	7.65	127.9	7.55	127.3	8.84	149.7
4	8.71	123.8	8.19	130.8	8.00	127.5	9.46	125.5	8.28	129.7	9.14	123.2	8.64	123.7	8.70	123.4		
4a		130.6		144.9		131.2		132.0		145.0		129.9		130.5		129.5		146.9
4b		130.7						142.4				129.2		130.7		131.0		
5	8.67	122.2			8.07	129.5					9.23	123.2	8.61	122.2	8.74	122.6	8.37	131.7
5a																		
6	7.73	127.7	9.10	144.2	8.15	128.8	9.23	142.5	9.39	144.0	8.48	129.2	7.65	127.7	7.78	128.0	8.50	133.1
6a				134.0		144.5		134.8		134.9		145.4						144.5
6b																		
7	7.73	127.2											7.65	127.2	7.76	127.3		
7a				143.9				144.5		142.1								
7b				127.5				127.5		128.1								
8	9.34	125.6	8.28	125.3	9.25	143.0	8.28	125.5	8.42	124.6	9.44	145.7	9.28	125.6	9.43	126.1	9.43	144.3
8a		131.1				135.1						136.6		131.1		131.2		136.7
8b		142.1												142.1		142.6		
9			7.63	127.3			7.64	127.3	7.63	126.4								
9a						144.7						144.5						144.9
9b						127.7						128.5						
10	9.07	142.5	7.70	128.1	8.39	125.6	7.70	128.2	7.57	127.1	8.55	124.3	9.08	142.4	9.24	141.9	8.10	121.3
10a		136.8												136.3		135.9		
11			8.76	123.6	7.69	127.3	8.77	123.6	7.82	128.9	7.76	126.7					8.16	131.5
11a		143.6		130.6				130.8		131.7				143.1		144.9		131.1
11b				129.9				129.8						128.4		127.8		
11c																		
11d																		
11e																		
12	7.97	123.6	8.76	124.0	7.75	128.4	8.76	124.0	7.14	123.5	7.73	127.3	8.27	124.9	8.41	125.9	8.10	129.3
12a																		
12b																		
12c																		
13	7.57	128.2	7.64	126.3	8.84	123.7	7.64	126.2	7.00	125.1	8.12	129.1	7.59	126.8	7.74	127.4	7.50	125.5
13a						131.1				132.5		132.4						
13b						129.0				137.5								
13c										119.2								
13d										125.5								
13e										130.1								
14	7.37	122.8	7.56	125.4	8.75	123.4	7.56	125.5	8.07	128.9	7.89	124.0	7.57	127.4	7.80	128.6	7.11	126.3
14a																		
14b																		
14c																		
15	8.82	126.8	8.90	126.9	7.48	125.8	8.82	127.2	6.94	125.9	8.83	124.2	7.91	129.0	8.84	123.9	7.51	128.1
15a		134.6		129.0				129.0				133.1		132.2		131.1		129.2
15b		136.1		129.7				129.4				137.1				129.2		129.8
15c		122.1		137.2				138.1				121.7						134.9
15d		127.8		124.2				121.6				126.3						121.6
15e																		123.5
16					6.96	125.4	8.66	124.2	7.45	125.8	9.13	127.2	7.65	124.2	8.75	123.5		
17					7.41	128.5	7.82	126.0	8.10	128.2	7.99	124.9	8.55	124.1	7.51	125.8		
17a						128.1		132.5		131.4		132.5		132.2				
17b						129.9								136.9				
17c						135.3								121.7				
17d						121.8								127.8				
17e						128.1												
18															7.03	125.5		
19															7.52	129.1		
19a																128.0		
19b																129.7		
19c																136.1		
19d																121.5		
19e																128.3		
